# The Historical Case for and the Future Study of Antibiotic-Resistant Scrub Typhus

**DOI:** 10.3390/tropicalmed2040063

**Published:** 2017-12-15

**Authors:** Daryl J. Kelly, Paul A. Fuerst, Allen L. Richards

**Affiliations:** 1 Viral and Rickettsial Diseases Department, Naval Medical Research Center, Silver Spring, MD 20910, USA; Allen.L.Richards.civ@mail.mil or allen.richards@comcast.net; 2 Department of Evolution, Ecology and Organismal Biology, The Ohio State University, Columbus, OH 43210, USA; fuerst.1@osu.edu

**Keywords:** scrub typhus, *Orientia*, *O. tsutsugamushi*, antibiotic resistance, antibiotic resistance genes, genome comparison

## Abstract

Scrub typhus is an acute, and sometimes fatal, human febrile illness, typically successfully treated using chloramphenicol or one of the tetracyclines. Over the past several years, descriptions of strains of *Orientia tsutsugamushi* with reduced susceptibility to antibiotics have appeared. Because case-fatality ratios approached 50% during the pre-antibiotic era, antibiotic-resistant scrub typhus is concerning. Herein, we review the data on resistant scrub typhus, describe how the theoretical existence of such resistance is affected by interpretation of treatment outcomes, and propose a plan to further identify whether true drug resistance is present and how to deal with drug resistance if it has evolved. Limited resistance is not unambiguous, if present, and antibiotic resistance in scrub typhus is not a dichotomous trait. Rather, evidence of resistance shows a continuous gradation of increasing resistance. The availability of genomes from isolates of *O. tsutsugamushi* allows the search for loci that might contribute to antibiotic resistance. At least eighteen such loci occur in all genomes of *O. tsutsugamushi* examined. One gene (*gyrA*) occurs as a quinolone-resistant form in the genome of all isolates of *O. tsutsugamushi.* At least 13 other genes that are present in some members of the genus *Rickettsia* do not occur within *O. tsutsugamushi.* Even though reports of scrub typhus not responding appropriately to chloramphenicol or a tetracycline treatment have been in the literature for approximately 23 years, the existence and importance of antibiotic-resistant scrub typhus remains uncertain.

## 1. Introduction

Scrub typhus (aka, tsutsugamushi disease or mite-borne typhus) is an acute, and sometimes fatal human febrile illness [1,2]. It is typically an easily treatable infection when recognized, but whereas chloramphenicol and the tetracyclines, and more recently azithromycin, are usually effective, common first-line antibiotics often used for the treatment of undifferentiated febrile illnesses, such as the beta-lactams (e.g., penicillin, cephalosporin), are not efficacious [1,3,4,5]. No licensed vaccine is currently available [1,2]. The disease is generally attributed to the bite of the parasitic chigger stage of vector *Leptotrombidium* sp. mites infected with the etiologic agent of the disease, *Orientia tsutsugamushi* (Rickettsiales: Rickettsiaceae). Interestingly, however, there have also been at least two unconfirmed reports of scrub typhus associated with leech bites [6,7]. Additional associated details are available in the Supplementary materials. Scrub typhus is endemic primarily within a 13,000,000-km^2^ roughly triangular region of the Asia-Pacific generally bordered by India-Pakistan, northern Australia and southern Siberia, a region often referred to as the ‘Tsutsugamushi Triangle’ [2,8]. Interest in this infection appears to be re-emerging as descriptions of strains of *O. tsutsugamushi* with reduced susceptibility to antibiotics are becoming common [9,10,11,12,13,14,15]. Also, there is evidence that the disease is increasing in incidence and spreading, with reports of confirmed cases in such disparate regions outside the traditionally-described Tsutsugamushi Triangle as Dubai, United Arab Emirates [16], Chile [7,17], Peru [18], and Africa [19,20,21,22,23,24,25,26]. Although more than a billion people are at risk, it is still generally considered a neglected tropical disease [8]. Nevertheless, interest and concern is growing rapidly. For example, in one analysis of scrub typhus publications identifying probable and confirmed cases, 60% of 145 publications screened were published since 2000 [8]. 

Improved diagnostic technologies and protocols, such as use of the highly-specific polymerase chain reaction, together with increased familiarity with the disease by clinicians even in remote locations, may at least partially explain the recent increase in reported cases [8,15,27].

Incidence appears to closely correlate with environmental disruption such as road construction, farming and plantation work, and more recently, recreational activities, such as ecotourism within endemic areas [10,28,29,30]. Scrub typhus has been seen as a moderate threat to deployed and training military personnel within the Asia-Pacific region [6,31,32,33]. *O. tsutsugamushi* is an obligate intracellular bacterium that parasitizes human or other vertebrate host endothelial cells lining capillaries, producing lesions, cell lysis, and a vasculitis within skin vessels and organs, inducing life-threatening shock [34]. A pathognomic lesion or eschar can appear at the site of the chigger bite but is often overlooked, especially in dark-skinned patients [35]. When left untreated, death often results from the resultant pneumonia, convulsions and hemorrhage. A recent comprehensive review of mortality data involving more than 19,000 untreated cases reported a 6.0% mortality rate, with a range of 0 to 70% [36]. Prior to the modern antibiotic era the disease often proved fatal, with variable mortality rates reported during World War II from 0.6% to as high as 55% in Yamagata, Japan [37]. During that era convalescence from the ‘severe’ form of the disease could last several months [6,38]. Although the new antibiotic penicillin was available, this was effectively the ‘pre-antibiotic’ era, especially for scrub typhus. In fact, in 1944 in one of the first demonstrations of intrinsic antibiotic resistance of scrub typhus to an antibiotic, penicillin was used in an attempt to treat scrub typhus patients subsequently proven to be isolate-positive. It was found to be completely ineffective [38,39,40]. 

Following the war, infection rates dropped dramatically because of anticipated reduction in non-wartime exposure, but also fatality rates plummeted with the successful introduction in 1948 of a new antibiotic, chloramphenicol (Chloromycetin; Parke Davis & Co., Detroit, MI, USA) [39]. United States Army researchers proved the efficacy of the drug in treatment of active scrub typhus [41]. Use as a prophylactic was marginal, however, since symptomatic reemergence or relapse of the suppressed infection was sometimes noted following discontinuation of the drug, suggesting possible resistance to the new drug [42]. In fact, Smadel reported recovering isolates from the lymph nodes of two chloramphenicol-treated scrub typhus patients about 4 months and 1 year after becoming afebrile [43]. Levels of antibiotic resistance associated with unrecognized recrudescence may be an overlooked aspect of scrub typhus, especially in geographic areas with high levels of endemic scrub typhus. Still, since the late 1940s this previously commonly fatal disease of the Asia-Pacific Rim has responded rapidly to antibiotic therapy. Assuming a case was recognized, scrub typhus was at long last under control. 

In 1953 the bacteriostatic protein synthesis inhibitor tetracycline was introduced, which in later trials proved to be superior to chloramphenicol. It produced a more rapid resolution of fever without the complicating blood dyscrasia sometimes reported for that antibiotic [34]. It was also found that tetracycline-treated patients became afebrile quicker than those treated with chloramphenicol. Tetracycline was used throughout the endemic area to treat the disease, including use during the war in Vietnam, in which scrub typhus was a common cause of febrile illnesses in allied forces [44]. A long-acting derivative of oxytetracycline, doxycycline, was patented in 1957 and FDA-approved in 1967 (Pfizer Inc., New York, NY, USA). As a lipophilic form of oxytetracycline, the drug was found to be rapidly absorbed from the gut and maintained in high levels in the blood. Doxycycline could be used either by mouth or intravenously and was found to be effective in both the treatment and prophylaxis of scrub typhus [45,46]. Timing of initiation of therapy proved important, however, in active cases of disease, relapse was particularly prominent (4 of 30, 13%) when treatment was initiated early, fewer than 5 days after onset of signs and symptoms [46]. The response was generally rapid with defervescence typically in 24 to 36 h. Failure to defervesce within 48 h could suggest an alternate diagnosis or even possible resistance [1,46]. Not surprisingly, since the antibiotic is bacteriostatic, recrudescence or relapse, not to be confused with resistance, was reported [46,47]. However, overall response to doxycycline therapy had been so dramatic that, with the lack of available, accurate and inexpensive diagnostic tests, physicians would use the rapid therapeutic response to confirm diagnosis. This was the case because the initial presentation of scrub typhus could appear similar to other endemic bacterial or viral febrile illnesses such as typhoid, leptospirosis, dengue fever, or even early malaria [35]. Hence, the typically consistent efficacy of chloramphenicol or tetracycline in treating scrub typhus was so powerful that Brown once suggested that, once a diagnosis of malaria had been excluded, an alternate diagnosis of typhoid should be considered most likely if there is failure to defervesce within 48 h [48]. However, the occurrence of antibiotic-resistant scrub typhus would negate such a conclusion.

## 2. Scrub Typhus and Antibiotic Resistance

### 2.1. Reports of Antibiotic-Resistant Scrub Typhus

Not surprisingly, the interest or concern of the busy clinicians for scrub typhus has been minimal, since for decades the treatment always worked and did so quickly. However, we appear to have entered what has been described as the ‘post-antibiotic’ era or the era of the ‘super-bug’ [49]. Reports of antibiotic resistance emanating from Thailand in the early 1990s and even more so in other regions in recent years, suggests that scrub typhus may prove to be no exception [9,10,11,12,13,14,15]. Because of the experiences of the pre-antibiotic era of World War II and the Asia-Pacific, when case-fatality ratios could reach 50%, even the hint of antibiotic-resistant scrub typhus has become of some concern. 

The historic and more recent impact of scrub typhus on the military of several countries has been well described [6,30,31,32,44,50]. Still, other than interest by the United States military due to potential impact on the fighting force due to lack of an effective vaccine and the obvious need to improve diagnostics, there appeared to be only moderate concern by the endemic medical community about this eminently-treatable disease. Some countries in the endemic area, such as Japan, did monitor the public health impact of scrub typhus, maintaining and publishing detailed case statistics, and performed correlating research on the vectors. Nevertheless, the prospect of antibiotic-resistant scrub typhus, while controversial, is supported by the evidence and has dramatically raised the profile of this disease.

It is the aim of this study to review the data on antibiotic-resistant scrub typhus, describe how the theoretical existence of such resistance is affected by the interpretation of treatment outcomes, and propose a plan of action to further identify whether true drug resistance is present and how to deal with drug-resistant scrub typhus if it has evolved.

### 2.2. Measuring Antibiotic-Resistant Scrub Typhus

*O. tsutsugamushi* has been shown to be intrinsically resistant to several classes of antibiotics including the penicillins, gentamycin, cephalosporins and, purportedly, the fluoroquinolones, although effective patient treatment has been reported with the latter [12,38,51,52]. While typically sensitive to antibiotics including chloramphenicol, doxycycline, tetracycline, rifampin, and the macrolides, including azithromycin, there exists a question about the interpretation of growing evidence of resistance to several of these drugs and how to detect that resistance in an organism previously found to be exquisitely sensitive. 

Since *O. tsutsugamushi* is an obligate intracellular bacterium, sensitivity testing can prove very difficult, labor-intensive and of arguable value due to lack of standardization. Intracellular antibiotic concentrations do not necessarily equate to serum levels [53,54]. There were no established methodologies that parallel the classic Kirby-Bauer method of sensitivity testing or the now-automated minimum inhibitory or minimum bactericidal concentration (MIC/MBC) determinations, in which in vitro growth is measured directly by instrumentation. New experimental methodology had to be developed. Hence, propagation of rickettsiae by inoculation of patient blood or infected animal tissues into embryonated hen eggs, cell culture, or animal inoculation and organ harvesting (murine) involving live *O. tsutsugamushi*, was required. These procedures call for biohazard level (BHL) 3 facilities, not often available to researchers or clinical laboratories [55]. In their preliminary resistance studies Watt et al. adapted previously-developed experimental methods to compare suspect clinically-resistant patient isolates with known sensitive strains [3,4,9,56,57]. Basically, a blood sample is taken from the febrile patient and inoculated into each of 2 adult ICR mice. When the mice become symptomatic, they are sacrificed, livers and spleens (L/S) aseptically excised, and impressions made for confirmatory staining. If positive, aliquots of the L/S suspension are prepared for further mouse inoculation and quantification by titration to determine the 50% murine lethal dose_,_ 50% tissue culture infective dose, plaque reduction quantification assay, or dye uptake assay, and prepared for antibiotic susceptibility testing [3,9]. Note that this procedure is performed for each suspected patient blood sample and can be quite labor intensive.

For MIC determinations, and since *O. tsutsugamushi* can only grow in cells, drug sensitivity testing can be performed in cell culture [3,12,56,57,58,59]. Again, the methodologies used have not been standardized, and minor variation in methodology is common. Slidechamber culture or multi-well cell culture plates are typically used. Irradiated L-929 mouse fibroblast cells (or other cell lines) are infected with rapidly-growing control organisms or a patient isolate. Rickettsiae are either inoculated directly onto plated host-cell monolayers or are first incubated with known concentrations of rickettsiae by tumbling them together with host cells to establish a uniform infection. Monolayers are rinsed, re-incubated with known concentrations of the test antibiotic, e.g., chloramphenicol, tetracycline, azithromycin, rifampicin or a fluoroquinolone, in cell culture medium and incubated for 3 days. Various antibiotic concentrations are tested in this manner, usually with at least one approximating that achievable in blood levels of treated patients. Following incubation, cells are rinsed, harvested and stained to quantify rickettsial growth by direct counting using light microscopy, plaque assay, or dye uptake [9,56,57]. In later studies, other means of particle quantification have been used, such as flow cytometry or DNA extraction of harvested cells, or molecular assays could be used to detect and quantify the rickettsiae [58]. Those methods include: standard PCR, nested PCR (nPCR), quantitative real-time PCR (qPCR) and loop-mediated isothermal amplification (LAMP) assays [12,27]. Drug concentrations are selected based on patient antibiotic levels achievable in vivo. This system gives quantifiable data with known sensitive strains (Karp, Kato, etc.) that can be compared directly within the same model. As stated, methodology has not been standardized, and assumes antibiotic concentrations are the same within the host cell as in the supernatant overlay [53]. However, we know that some antibiotics show poor penetration of host cell membranes, whereas other antibiotics are actually concentrated within the cell [60]. Future studies might address this quandary by direct measurement of intercellular antibiotic levels. However, defining the actual MICs that correlate with complete or intermediate susceptibility, or real resistance to the evaluated antibiotics, remains a problem. Such determinations can depend on the patient, antibiotic used, the organism itself, or even the competing vested interests of the drug developer/manufacturer [54]. 

A murine model has also been used to monitor resistance [51]. Using this model, morbidity, mortality and change in body weight of the inoculated, antibiotic-treated mouse is compared with a control antibiotic-susceptible strain (Karp) [3,9]. However, the validity of this model to examine resistance, including the proper antibiotic dosages to administer, remains questionable [12]. Still, it is a live, practical model. 

In vitro and in vivo fever clearance times between groups of antibiotic-treated patients, the time between the first dose of the antibiotic and the patient’s return to normal temperature (=37 °C), is one tested method to detect antibiotic resistance [9]. Typically, a febrile scrub typhus patient should defervesce within 48 h following initiation of appropriate therapy. Establishing the number of days of fever before admission/treatment is very important, since death or clinical presentation following delayed treatment might erroneously appear to be caused by a resistant strain. 

Finally, qPCR of patient blood samples can be used directly to measure efficacy of treatment. However, the antibiotics used to treat scrub typhus are bacteriostatic and positive PCR results have been found in afebrile patients who have been successfully treated [27].

### 2.3. Reports of Human Antibiotic Resistance

Patient relapses and persistence of scrub typhus rickettsiae in tissues of recovered patients have been reported with both chloramphenicol and doxycycline therapy [43,46,47]. However, one of the earliest indications of actual antibiotic resistant scrub typhus was proposed in the early 1990s, based on the observations by physicians, who were seeing febrile patients in northern Thailand [9]. Profoundly-ill scrub typhus patients were identified, who were shown to be non-responsive to appropriate antibiotic therapy. Although there was initial doubt about the etiology, rickettsial isolation and serology suggested it to be a previously-unreported antibiotic-resistant form of infection.

A local physician, Dr. Charoen Chouriyagune of the Chiangrai Prachanuchroa Hospital, and Dr. George Watt, a United States Army clinical researcher assigned to the Armed Forces Research Institute of the Medical Sciences, Bangkok (AFRIMS), observed presumptive scrub typhus patients presenting at the hospital failing to respond to oral doxycycline or intravenous chloramphenicol treatment, and demonstrating a mortality rate as high as 15% [9,61]. Based upon those early observations, a small prospective clinical trial was conducted, comparing select patient populations from Mae Sot, western Thailand (*n* = 7), with patients from the Chiangrai region (*n* = 12). Chiangrai cases were confirmed by isolation of rickettsiae and seroconversion. Their study included: (1) the direct comparison of the two patient populations for fever-clearance times; (2) survival rates of doxycycline- or chloramphenicol-treated mice, following inoculation with Chiangrai patient-derived *Orientia* isolates (*n* = 3); and, (3) in vitro determination of the percentage of cells positive in murine L-929 cell culture following infection with patient-derived rickettsiae. The results suggested the following: (1) mean fever clearance times were significantly greater in the Chiangrai patients than Mae Sot patients; (2) there were significantly more deaths in resistant strain-infected mice treated with chloramphenicol, in contrast to the control organism; and (3) although all 3 isolates were susceptible to chloramphenicol, one isolate showed diminished susceptibility to both antibiotics but clear resistance to doxycycline. The data showed this strain, identified as C3 (now labeled AFC3), to be clearly the most ‘antibiotic-resistant’ strain. In an effort to repeat these findings using the murine model and possibly identify an alternate antibiotic treatment for the doxycycline-resistant strains, the susceptibilities of those 3 strains were tested using azithromycin or doxycycline [4]. In contrast to the complete efficacy (100% survival) of control Karp strain-infected mice, antibiotic treatment of C3 (63%) and C27 (75%) infected mice were only marginally effective, again suggesting at least partial doxycycline resistance. Some clinical evidence suggests this resistance phenomenon is not focused within the Chiangrai region alone. In a 1990 clinical study by the Royal Thai Army, an *O. tsutsugamushi* isolate was recovered from a patient in Kanchanaburi Province, western Thailand, more than 500 km from Chiangrai. Identified as AFSC4, it appeared in later in vitro studies to have ‘sharply reduced susceptibility to doxyxcycline’, and was described as a ‘doxycycline-resistant strain’ [3,13,61]. Those studies introduced the new macrolide, azithromycin, to scrub typhus research. The bacteriostatic agent appeared to be a potential alternative to doxycycline and suppressed in vitro growth of *O. tsutsugamushi,* including the newly-identified resistant strain. It also had the benefit of being an optimal substitute for the treatment of pregnant women, in which the tetracyclines or chloramphenicol are contraindicated, and children, in which tetracycline treatment is not recommended [3,5]. Their assay used percentage of infected cells and rickettsiae per cell as monitors of rickettsial growth. They found AFSC4 to be significantly more resistant to doxycycline than the susceptible control Karp strain. A later prospective treatment trial conducted in Korea supported the efficacy of azithromycin in the treatment of scrub typhus in patients as a possible alternative to doxycycline [5]. 

**Figure 1:**
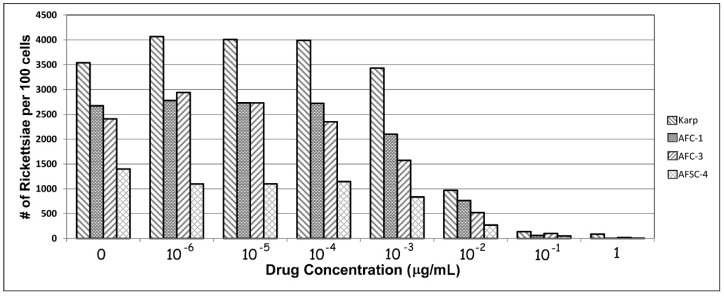
In vitro responses of isolates of *O. tsutsugamushi* to various levels of doxycycline following 72 h of incubation.

Still, these findings of antibiotic resistant *O. tsutsugamushi,* as noted above, were, not surprisingly, quite controversial. In order to confirm their findings, we repeated in vitro portions of the studies of Watt and Strickman using two Chiangrai isolates: C1 and C3, the purportedly antibiotic-resistant strains, and AFSC4, the Kanchanaburi resistant strain, using the Karp strain as a control. The AFSC4 strain had been selected in the earlier study based upon reduced susceptibility to doxycycline, but was otherwise not described [3,9]. Figure 1 shows our results for the four strains. Although rickettsial growth is not suppressed in any of the strains at the greatest antibiotic dilutions tested (1 × 10^−6^ µg/mL), growth is inhibited at (0.1 µg/mL), corresponding to published MICs. In this in vitro model, growth is suppressed for all 4 strains at achievable blood levels of doxycycline concentrations equivalent to MICs typically achieved in vivo in patient therapy [59]. At least in this model, resistance appears illusory.

Since the paper by Watt et al. there have been additional cases reported, suggesting antibiotic-resistant scrub typhus [9]. The Watt research group continued the effort to identify *bona fide* resistant strains and to evaluate alternative therapies for use in regions where doxycycline continued to be marginally effective. They noted two relapses after doxycycline therapy, but none after rifampicin therapy [47]. The rifampicin-doxycycline treatment trial completed in 1997 in Chiangrai included patients showing a wide variety of responses to the test antibiotics. It was an extensive trial in which more than 12,000 febrile patients were screened, resulting in 126 patients with confirmed scrub typhus and no other infection. Investigators found that fever clearance times in proven scrub typhus patients were significantly shorter with rifampicin treatment than doxycycline. That trial also resulted in the recovery of more than 100 patient isolates of *Orientia*. Fifteen of those 60 isolates (SV-series) were further tested at the United States Naval Medical Research Center against 5 antibiotics, to identify the most resistant strains. The results of this study were reported in an unpublished Executive Summary Statement in 1998 from the Rickettsial Diseases Research Program (STEP J of the Military Infectious Diseases Research Program) of the United States Army Medical Research and Material Command. Unfortunately, the associated clinical records were unavailable to correlate patient response to the antibiotic therapies. In addition, as reported in this executive summary, an isolate recovered from Mae Sot, labeled KR-3, in which the patient was known to have delayed response to antibiotic therapy, was identified as a potential antibiotic resistant strain. 

More recently, in addition to those earlier reported cases and studies, multiple treatment failures and deaths were reported in antibiotic-treated scrub typhus patients in north-eastern Thailand [15]. In the study, 146 patients had confirmed scrub typhus upon admission to Maharat Nakhon Ratchasima Hospital, June to December 2010. Of these, there were 9 (6.2%) fatal *O. tsutsugamushi* PCR-positive or seropositive cases reported, 6 of which received chloramphenicol or doxycycline. Investigators suggested treatment failures were due to doxycycline resistance in the 3 fatal cases receiving doxycycline. 

In addition to the earlier focus in northern Thailand, there have been some reports of possible antibiotic-resistant scrub typhus outside of Thailand. Due in part to those earlier reports, investigators may have become sensitized to the possibility of antibiotic resistance. Corwin reported 14 scrub typhus seroconversions in 347 individuals (mostly United States military personnel) deployed to scrub and forested terrain within Laos (*n* = 5) and Vietnam (*n* = 9) between June 1996 and April 1998 [31]. The personnel ‘without exception’ received mandatory doxycycline malaria prophylaxis, 100 mg/day, during deployment. In addition to seroconversion, 3 of the 14 presented with signs and symptoms of typical scrub typhus. These data are certainly suggestive of doxycycyline-resistant scrub typhus, since prophylaxis failed to prevent the breakthroughs. 

Lee et al. reported ‘doxycycline resistant’ *O. tsutsugamushi* in an initially IgM-seropositive, eschar-positive, meningoencephalitis patient in South Korea [14]. The farmer failed to defervesce following initiation of oral doxycycline, but responded within 3 days of the initiation of oral azithromycin therapy. Timing of the initiation of therapy can be crucial to the successful treatment of scrub typhus but may have a questionable role in resistance. 

Misdiagnosis with delayed appropriate treatment can prove fatal. For example, Singh reported a soldier in India initially treated for malaria and later, following discovery of an eschar, treated with chloromycetin in his 9^th^ day of fever [62]. The soldier died of the scrub typhus infection the 10th day of fever. In another case of delayed treatment, Mathai et al. speculated that doxycycline resistance may have played a role in a serologically-confirmed fatal case in southern India [11]. The patient, who had been febrile for 10 days, initially defervesced following start of doxycycline treatment, but died a few days later following septic shock. Failure due to delayed treatment in these cases might suggest doxycycline resistance, but correct therapy might simply have been started too late. 

### 2.4. Theoretical Aspects of Antibiotic-Resistant Scrub Typhus

There still appears to be some doubt about the existence of resistance in spite of published clinical, in vitro, and in vivo confirmatory data. It is not apparent how selection for such resistance could occur. Is acquired antibiotic resistance by the infecting organism, i.e., evolution of antibiotic resistance, a possible explanation for patients becoming infected with resistant strains and thus not responding to antibiotic therapy? In theory, these examples of what appear to be antibiotic resistance appear paradoxical and are unlikely to reflect the evolution of antibiotic resistance because the mechanism of selective antibiotic pressure is unapparent. The vector mite feeds but once on a vertebrate in its lifecycle, and laboratory-based attempts to infect mite lines from infected rodents have generally (but not always) failed. Most infected colony-based attempts to produce infected, F-1 chiggers by feeding the larvae of the parental generation on infected rodents have failed [63,64]. However, there is at least one case in which offspring maintained an infection. Traub et al. reported the persistence of acquired *O. tsutsugamushi* in mite offspring for up to 2 weeks and a single case of transovarial transmission to a subsequent generation [65]. Although evidently rare, this single case demonstrates the potential for an uninfected mite to bite a rickettsemic host, acquire a possibly antibiotic-resistant strain and subsequently pass it on to other reservoirs. Thus, the mite appears to have the potential to be both the reservoir and the host leaving rodents or humans as dead-end hosts. 

It is also possible that this antibiotic resistance may have been present in the population, naturally, and only relatively recently have clinicians, armed with better diagnostic tools, begun to recognize such cases in any depth. There is recent evidence, garnered from ancient permafrost sediments, isolated caves, and in human specimens preserved for hundreds of years, of identified genes coding for tetracycline resistance that would not have been subjected to selective pressure [66,67,68]. Other evidence for a mechanism that could allow the non-selective acquisition of resistance also exists, such as recent results suggesting that non-selective mechanisms in *Orientia,* including homologous recombination, may be in play [69].

However, there are also some other aspects of the biology of *O. tsutsugamushi* that may play a role in apparent antibiotic resistance. There may be a role for virulence factors, which could render one strain more virulent than another. Hence antibiotic treatment might not be timely, and the patient might succumb to an infection not because of antibiotic resistance, but rather, because the patient faces a more virulent organism. Some strains of *O. tsutsugamushi* may code for proteins or virulence factors released into infected cells. There may not be an antibiotic-resistance gene, but rather a gene coding for rapid growth in vivo for example. Still, such genes would appear in the DNA of one strain and not in others, and hence should be identifiable in whole genome comparisons. One example might be a gene that would produce a toxin, such as that found in the Gilliam strain of *O. tsutsugamushi*, that thus far has not been observed in other strains. This might produce a life-threatening toxic effect, independent of the antibiotic therapy [40,70,71]. The concept of virulence is a difficult one to quantify. The Gilliam strain of *O. tsutsugamushi* demonstrates this. Except for select inbred mouse strains, Gilliam is not virulent in mice, which was used as a reason to consider it as a potential vaccine candidate. However, in humans it can be highly virulent (as illustrated by the fact that it almost killed Andrew Gilliam, a United States Public Health Service officer-physician working in Assam, India, when he contracted scrub typhus in December 1943) [6,40]. 

There is also some evidence that there is a seasonal pattern in the occurrence of scrub typhus in many endemic areas, such as China, Korea and Japan [72,73,74]. Within this seasonality, there is also the suggestion that scrub typhus may show differences in the severity of symptoms and higher mortality between seasons [73,74]. Is this an indication that some isolates, associated with different vectors, may be more virulent? This seems to be an open question. 

### 2.5. Genomics of Antibiotic Resistance

One approach to searching for the basis of antibiotic resistance in *Orientia* would be to examine whole or partial genome sequences that are now becoming increasingly available for study. There are currently whole genomic sequences, or whole genome contigs, available from 12 isolates of *O. tsutsugamushi* and from *Candidatus* Orientia chuto [75,76,77,78,79]. In addition, 26 additional isolates have been studied for sequences that will soon appear as whole genome sequences, but which are currently associated with sequence read archives (SRA) [80]. The SRA files are available for study in the DNA databases. Almost all of the isolates that have been studied for genome sequencing are described in an appendix in reference [80]. 

Two methods can be used to utilize this information from almost 40 genomes. In the most direct approach, we can search for sequences within the genome of isolates of *O. tsutsugamushi* that have homology with genes known to facilitate antibiotic resistance in other organisms. Information concerning how many such genes occur in the genome and knowledge of how they play a role in other bacteria can guide us to evaluate their importance in potential resistance in contemporary foci of scrub typhus. The second procedure can be used when we have genomic information available for isolates that display the characteristics of antibiotic resistance. To apply this procedure, we would compare the entire genomic sequences of antibiotic-resistant isolates with the complete genomic information from non-resistant isolates. We have begun an examination of both these approaches. 

### 2.6. Search for Specific Genes That Can Contribute to Resistance

The whole genome sequences of 12 *O. tsutsugamushi* and the *Ca.* O. chuto isolate have been examined for the presence of putative resistance genes. This examination has led to several conclusions. It is clear that a number of such genes appear to be present in the genomes of most, probably all, isolates of *O. tsutsugamushi,* as well as in *Ca.* O. chuto.

The genes or gene categories that may have the potential to affect levels of resistance to antibiotics include the following classes that we have found to be present in the genomes of *Orientia* isolates: (1) Bcr/CflA drug resistance efflux transporters (at least two loci); (2) ABC-type multidrug transport system (at least 5 loci); (3) RND family efflux transporters (the locus of at least one component gene); (4) penicillin-binding proteins (at least 3 loci); (5) beta-lactamases (at least 2 genes); (6) the ampG1 permease of the major facilitator superfamily (1 locus); (7) microcin C resistance proteins (1 locus); and (8) DNA gyrase (1 locus, the *gyrA* locus). Furthermore, there can be additional genes such as those involved in resistance to macrolide, lincosamide, and streptogramin (MLS) antibiotics that affect RNA or DNA synthesis and that have known resistance mutations in other bacteria. At least two such loci can be found in the genomes of *Orientia*. These yield a total of at least 17 genes that have the potential to contribute to any antibiotic resistance that may occur in isolates of *Orientia.* Further details, including the locations of specific loci in the genomes of the Boryong and Ikeda isolates can be found in the Supplementary Materials. 

Several features of these loci characterize their existence in the genomes of *Orientia*. First, all of the loci mentioned above exist within all 13 genomes of *Orientia* that we have examined in detail. We hope to examine the genomes of the 26 isolates that currently exist only as SRA files in the near future. Second, all of the loci display sequence variation between isolates at both the nucleic acid and the amino acid levels. This makes the evaluation of their potential to mediate antibiotic resistance difficult. Unless we have specific information that allows us to target a particular sequence element, we would be unable, from sequence information alone, to predict the effect of particular mutations. In general, differentiation between isolates (at both the DNA and protein levels) follows very roughly the expected patterns predicted by some other genes that have been used for phylogenetic analysis and that have no association with potential antibiotic resistance. 

Note that two of the isolates for which genome sequences have been determined are AFSC4 and AFSC7, isolates that we discussed above. These are isolates that have putatively shown some level of antibiotic resistance. The nucleic acid sequences for all of the loci that we have examined from isolates AFSC4 and AFSC7 show differences compared to other isolates. However, no unusual patterns were seen in which a sequence from either isolate was clearly more divergent than expected. Such extreme divergence might be suggestive of an unusual pattern of selection on a locus in these isolates compared to the role played by the locus in other sensitive isolates. 

There is one gene for which we can examine a predicted effect of a specific mutation. This is the *gyrA* gene (coding for the protein DNA gyrase). Ciprofloxacin, a member of the fluoroquinolones, is known to inhibit bacteria due to interaction with bacterial DNA gyrase. Mutations in *gyrA* gene in *Bartonella* were shown to be related to failure of treatment by ciprofloxacin. In *Orientia*, Tantibhedhyangkul et al. showed that the *gyrA* gene in the Kato isolate of *O. tsutsugamushi* contained an amino acid homologous with that found in the *gyrA* gene of *Bartonella* (a Ser83Leu mutation in the QRDR domain of *gyrA*) [12]. They also showed that this amino acid configuration occurred in isolates from Laos. 

We have examined the *gyrA* gene in genome sequences of all 38 isolates of *O. tsutsugamushi* and in *Ca.* O. chuto, as well as a representative sample of genomes from other members of the Rickettsiaceae. In all isolates of *O. tsutsugamushi* and *Ca.* O. chuto, leucine was the amino acid found at position 83 (Leu83) of the QRDR domain of *gyrA*. In contrast, no other member of the Rickettsiaceae was found to possess leucine at this position. All members of the genus *Rickettsia* that we examined had serine at this position. *Neorickettsia* also had serine, while *Anaplasma* isolates had threonine. *Ehrlichia* isolates had alanine. *Wolbachia* isolates had methionine. Finally, more distantly-related intracellular Rickettsiales generally had serine. The occurrence of this resistance seems to be uniquely a characteristic of *Orientia*, and must have occurred very early after the divergence of *Orientia* from the ancestor it shared with *Rickettsia*. 

Other potential genes of interest also exist in the genomes. Many antibiotics work by inhibiting interactions between ribosomal RNAs and ribosomal proteins. In *Rickettsia*, differences in drug susceptibility between the typhus and spotted fever groups of species appear to be related to genetic changes in the large subunit rRNA (23S rRNA) gene, together with changes at a ribosomal protein (L22) [81]. Both the gene for L22 and for the 23S rRNA show variation between isolates of *Orientia*, but none of this variation can be implicated in any possible antibiotic resistance, either in isolate AFSC4 or in other isolates. Other potential genetic sources of antibiotic resistance in members of *Rickettsia* have been identified and we can assume that a careful comparison of well-curated genome sequences from various isolates of *O. tsutsugamushi* will yield additional insights [82]. When comparing our analyses with those in *Rickettsia*, it appears that *Orientia*, despite having a larger genome and a larger number of potential genes (ORFs), seems to have fewer elements that can be identified as having a putative potential for effecting antibiotic resistance. In every one of the classes of loci that may affect potential antibiotic resistance, *Orientia* has either the same or fewer loci that can be identified compared to many or most species of *Rickettsia*. In fact, we have identified at least 13 specific loci that are present in most or all members of *Rickettsia* that we examined, but are absent in the genome sequences of *Orientia*.

As mentioned, there is a second method to assess potential antibiotic resistance in a genome. This is a direct comparison of the genomes of isolates that evince antibiotic resistance with those of sensitive isolates. Such comparisons can produce a list, however small, of loci that are present in the resistant isolate that may cause resistance. In theory, this should provide a definitive answer. Such a comparison has been reported as a first attempt [78]. Liao et al. did a direct comparison of the genome sequences of the Karp isolate (a sensitive strain) with the genome sequences of AFSC4 and AFSC7. They reported the discovery of two ORFS (labeled gene 1436 and gene 2073) that were present in both AFSC4 and AFSC7 but absent in Karp. We have examined these two genes in the other whole genome sequences of *Orientia*. 

Our examination indicates that gene 1436 is present in both AFSC4 and AFSC7, but was also present in Karp (assessed by examination of the SRA of Karp in the DNA databases). Gene 1436 was also present in the genomes of the isolates Ikeda, Kato, UT76, UT144, TA716 and TA763 (all considered sensitive to chloramphenicol). The locus was absent in isolates Gilliam, Boryong, Sido and *Ca.* O. chuto.

For gene 2073, our studies show that the locus is present in AFSC4 and AFSC7. In the Karp isolate, the locus appears to have been non-functionalized due to a number of mutational changes, which makes identification more problematic. However, the locus is present and apparently intact in chloramphenicol-sensitive isolates Kato, Ikeda, UT76, and TA763. It has accumulated a single non-functualizing mutation in the Gilliam isolate. It appears to be defective/decayed, but present in isolate TA716. We find no evidence of a strongly similar sequence in UT144, Sido or *Ca.* O. chuto.

These results indicate the difficulty of making whole genome sequence comparisons with isolates of *Orientia*. There are long repetitive sequences that have made the sequencing of isolates of *Orientia* a challenge. The genomic data for isolates of *Orientia* must be examined with great care to conclude that a gene is either missing or newly appearing and not present in other strains.

### 2.7. Horizontal Transfer of Resistance

Genetic recombination in *Orientia* presents a challenge to the approach of searching for the existence of specific resistance genes in putative drug-resistant isolates. A straightforward approach would compare genome sequences from drug-resistant and drug-sensitive strains, to search for genetic differences between sensitive and resistant genomes. Recombination, possibly associated with the large proportion of the genome composed of repetitive elements, is suspected to occur much more frequently in *Orientia* than in other members of the Rickettsiales. There is the possibility that isolates may acquire resistance through horizontal transfer from other isolates or from other bacteria. One problem with the comparative approach for the study of scrub typhus is that *Orientia* genes/genomes show substantial variability within the genus. This variability includes nucleotide differences, differences in insertion or deletion (in/del) of sequences, differences in the number of repeat sequences, and differences in the order and location of genes within a ‘standard’ template of the genome of the species *O. tsutsugamushi*.

To successfully identify a gene or site that contributes to resistance, comparisons must be made with isolates that are very similar genetically to putative resistant strains. While routine comparisons of specific genes can usually be performed in a straightforward manner, comparisons of whole genomes are much more difficult. Currently, the sizes of genome assemblies of various isolates of *Orientia* available for comparison are highly variable. The two genomes for which a ‘completed’ sequence has been reported, isolates Boryong and Ikeda, are fairly similar in size (2,127,051 bp and 2,008,987 bp, respectively). For twelve other isolates the genome information does not currently consist of a closed genome sequence. It is assumed that the existence of highly-repeated sequences within the genome prevent the exact order of genes to be determined. However, size of the genome assemblies for these twelve isolates is quite heterogeneous (Table 1). 

The genome assemblies range from a small of 712,858 bp (Sido), up to 3,033,399 bp for UT76. The two versions of Karp represent independent sequences of the same original isolate, but these efforts have currently resulted in substantially different total basepairs being reported for the genome assemblies. This cannot be true. It must be the result of difficulties in resolving contigs. It is possible that the size of the assemblies, shown in Table 1, correctly reflects the size of the genome. However, it is also possible (in our opinion more likely) that differences simply represent difficulties in linking sequence fragments into larger contigs and eventually into a completed genome. Sequence fragments not currently included in the genome assemblies would be considered a missing gene, but may simply reflect the lack of recognition of a sequence. These problems in the assembly of the completed genome make the assessment of horizontal transfer more difficult. 

As we continue to accumulate information on genome sequences, if a gene or genes appear to be missing from the genome of an isolate, it is imperative that these parts be carefully screened onto the array of sequence fragments (for instance onto the Sequence Read Archive) of the isolate. This will allow the identification of any sequences that are actually present, but which appear initially to be missing from completed contigs. 

Given the difficulties of comparing genome sequences within *Orientia*, an alternative supplementary approach to the search for sequence differences significant to antibiotic resistance may be possible, especially if more than one of the putative resistant isolates are actually very different genetically from each other and from other isolates. In this case, a comparison between the putative resistant isolates, searching for sequences which show unusual similarity (compared to other genomes), might provide possible target genes.

## 3. Discussion/Conclusions

We believe determination of the existence of antibiotic-resistant scrub typhus is an important public health question, especially within the ever-expanding endemic area of the disease. In our opinion the extant data reviewed here remain inconclusive or show only weak support for the case of acquired antibiotic-resistant scrub typhus. In spite of in vivo, in vitro and clinical data the question of the existence of true antibiotic-resistant scrub typhus remains. Resistance in general simply does not appear to be an ‘all or none’ phenomenon. In the case of ‘antibiotic-resistant’ scrub typhus this appears so. Strains isolated so far from patients who do not respond to therapy, while not thriving in the presence of the antibiotics, still manage to grow and compromise the patient. However, data do suggest further clinical trials and laboratory-based studies are needed to definitively make the case for or against resistance, and importantly, to establish acceptable, reproducible breakpoints, as well as acceptable treatment protocols that can be initiated as soon as initial treatment failure is discovered. 

The use and validation of MICs and breakpoints to establish which *O. tsutsugamushi* isolates are antibiotic resistant can be problematic. Diene and Raoult point out the complexity and arbitrary nature of the definition of drug resistance in general and especially in obligate intracellular pathogens [54]. Breakpoints can be artificial and influenced by human opinion that can be both politically and financially driven. In addition to the isolate itself, overall health of the patient, individual patient response to therapy, the antibiotic used, and even the proprietary nature of the treatment all represent complex issues. 

As our genetics capabilities expand and ever more whole-genome sequences are completed, genes that code for whole or partial resistance may be identified. Even so, there have recently been reported mechanisms of resistance that are not necessarily acquired or are transmissable genetic elements. With this in mind, renewed efforts are underway to identify non-antibiotic-responsive cases with correlating efforts to isolate viable resistant strains. The genomic analyses that can currently be performed do not yet provide any information about the existence of mutations in *O. tsutsugamushi* that would mediate resistance to the traditional antibiotic regimen. Genomic studies of additional isolates that show the possibility of antibiotic resistance are obviously necessary for this to change.

Clearly, the small study of Watt et al. should be repeated or expanded, both in Chiangrai, as well as at control sites [9]. An effort should also be made to identify sites outside of Thailand, especially where other antibiotic-resistant cases have been reported, in order to initiate the study of the distribution of resistant cases. It is important that the product of such a study should demonstrate whether resistance to antibiotics by the agent of scrub typhus was the cause of death, or whether the specifics of patient treatment (prior health of patient, time to diagnosis, time and length of antibiotic treatment, etc.) produced the negative outcome.

## Figures and Tables

**Table 1:** Genome Sizes of Completed Genomes and of Genome Assemblies of Isolates of *Orientia*.

Isolate Name	Whole Genome Sequence Project	# of Contigs	Size (bp)
Boryong ^1^	NC_009488	1	2,127,051
Ikeda ^1^	NC_010793	1	2,008,987
AFSC4	LYMT01	452	1,295,323
AFSC7	LYMB01	485	1,437,566
Karp	LYMA02	108	2,026,724
Karp	LANM01	145	1,454,354
UT144	LAOR01	229	1,689,193
Sido	LAOM01	83	712,858
TA716	LAOA01	234	2,221,260
UT76	LANZ01	332	3,033,399
TA763	LANY01	194	2,460,104
Gilliam	LANO01	76	1,997,698
Kato PP	LANN01	137	1,478,442
*Ca.* O. chuto	LANP01	47	1,092,196

^1^ Completed genomes; isolate descriptions in [80].

## Supplementary Materials

Additional associated general details are available online at https://u.osu.edu/crubtyphus; details about antibiotic resistance genes are available at https://u.osu.edu/scrubtyphus/ntibiotic-resistance-genes/.

## References

[1] Kelly D.J. (1999). *Orientia* *tsutsugamushi*. Antimicrobial Therapy and Vaccines.

[2] Kelly D.J., Fuerst P.A., Ching W.-M., Richards A.L. (2009). Scrub typhus: The geographic distribution of phenotypic and genotypic variants of *Orientia tsutsugamushi*. Clin. Infect. Dis..

[3] Strickman D., Sheer T., Salata K., Hershey J., Dasch G., Kelly D., Kuschner R. (1995). In vitro effectiveness of azithromycin against doxycycline-resistant and susceptible strains of *Rickettsia tsutsugamushi*, etiologic agent of scrub typhus. Antimicrob. Agent Chemother..

[4] Watt G., Kantipong P., Jongsakul K., Watcharapichat P., Phulsuksoombati D. (1999). Azithromycin activities against *Orientia tsutsugamushi* strains isolated in cases of scrub typhus in northern Thailand. Antimicrob. Agents Chemother..

[5] Kim Y.-S., Yun H.-J., Shim S.K., Koo S.H., Kim S.Y., Kim S. (2004). A comparative trial of a single dose of azithromycin versus doxycycline for the treatment of mild scrub typhus. Clin. Infect. Dis..

[6] Jones W.S., Stone J.H. (1969). Chinese liaison detail. Crisis Fleeting.

[7] Balcells M.E., Rabagliati R., Garcia P., Poggi H., Oddo D., Concha M., Abarca K., Jiang J., Kelly D.J., Richards A.L. (2011). Endemic scrub typhus-like illness, Chile. Emer. Infect. Dis..

[8] Kelly D.J., Foley D.H., Richards A.L. (2015). A spatiotemporal database to track human scrub typhus using the VectorMap application. PLoS Negl. Trop. Dis..

[9] Watt G., Chouriyagune C., Ruangweerayud R., Watcharapichat P., Phulsuksombati D., Jongsakul K., Teja-Isavadharm P., Bhodhidatta D., Corcoran K.D., Dasch G.A. (1996). Scrub typhus infections poorly responsive to antibiotics in northern Thailand. Lancet.

[10] Watt G., Parola P. (2003). Scrub typhus and tropical rickettsioses. Curr. Opin. Infect. Dis..

[11] Mathai E., Rolain J.M., Verghese G.M., Abraham O.C., Mathai D., Mathai M., Raoult D. (2003). Outbreak of scrub typhus in southern India during the cooler months. Ann. N. Y. Acad. Sci..

[12] Tantibhedhyangkul W., Angelakis E., Tongyoo N., Newton P.N., Moore C.E., Phetsouvanh R., Raoult D., Rolain J.-M. (2010). Intrinsic fluoroquinolone resistance in *Orientia tsutsugamushi*. Int. J. Antimicrob. Agents.

[13] Kim M.S., Baek J.H., Lee J.-S., Chung M.-H., Lee S.M., Kang J.-S. (2013). High in vitro infectivity of a doxycycline-insensitive strain of *Orientia tsutsugamushi*. Infect. Chemother..

[14] Lee S.-H., Chung E.J., Kim E.-G., Sea J.H. (2014). A case of doxycycline-resistant tsutsugamushi meningoencephalitis. Neurol. Asia.

[15] Thipmontree W., Tantibhedhyangkul W., Silpasakorn S., Wongsawat E., Waywa D., Suputtamongkol Y. (2016). Scrub typhus in northeastern Thailand: eschar distribution, abnormal electrocardiographic findings and predictors of fatal outcome. Am. J. Trop. Med. Hyg..

[16] Izzard L., Fuller A., Blacksell S.D., Paris D.H., Richards A.L., Aukkanit N., Nguyen C., Jiang J., Fenwick S., Day N.P. (2010). Isolation of a novel *Orientia* species (*O. chuto* sp. nov.) from a patient infected in Dubai. J. Clin. Microbiol..

[17] Weitzel T., Dittrich S., López J., Phuklia W., Martinez-Valdebenito C., Velásquez K., Blacksell S.D., Paris D.H., Abarca K. (2016). Endemic scrub typhus in South America. N. Engl. J. Med..

[18] Kocher C., Jiang J., Morrison A.C., Castillo R., Leguia M., Loyola S., Ampuero J.S., Cespedes M., Halsey E.S., Bausch D.G. (2017). Serologic evidence of scrub typhus in the Peruvian Amazon. Emerg. Infect. Dis..

[19] Giroud P., Jadin J. (1951). The presence of antibodies against *Rickettsia orientalis* in indigenous and Asian (peoples) living in Ruanda-Urundi (Belgian Congo). Bull. Soc. Pathol. Exot..

[20] Osuga K., Kimura M., Goto H., Shimada K., Suto T. (1991). A case of tsutsugamushi disease probably contracted in Africa. Eur. J. Clin. Microbiol. Infect. Dis..

[21] Ghorbani R.P., Ghorbani A.J., Jain M.K., Walker D.H. (1997). A case of scrub typhus probably acquired in Africa. Clin. Infect. Dis..

[22] Groen J., Dolmans W., Ligthelm R.J. (1999). Scrub and murine typhus among Dutch travellers. Infection.

[23] Cosson J.F., Galan M., Bard E., Razzauti M., Bernard M., Morand S., Brouat C., Dalecky A., Ba K., Charbonnel N. (2015). Detection of *Orientia* sp. DNA in rodents from Asia, West Africa, and Europe. Parasites Vectors.

[24] Thiga J.W., Mutai B., Eyako W., Ng’ang’a Z., Jiang J., Richards A.L., Waitumbi J.N. (2015). High sero-prevalence and IgG titers for spotted fever and scrub typhus in patients with febrile illness in Kenya. Emerg. Infect. Dis..

[25] Horton K.C., Jiang J., Maina A., Dueger E., Zayed A., Ahmed A.A., Guillermo Pimentel G., Richards A.L. (2016). Evidence of *Rickettsia* and *Orientia* infections among abattoir workers in Djibouti. Am. J. Trop. Med. Hyg..

[26] Maina A.N., Farris C.N., Odhiambo A., Jiang J., Laktabai J., Armstrong J., Holland T., Richards A.L., O’Meara W.P. (2016). Q fever, scrub typhus, and rickettsial diseases in children, Kenya, 2011–2012. Emerg. Infect. Dis..

[27] Luce-Fedrow A., Mullins K., Jiang J., Richards A. (2015). Strategies for detecting rickettsiae and diagnosing rickettsial diseases. Future Microbiol..

[28] Brown G.W., Robinson D.M., Huxsoll D.L. (1976). Scrub typhus: A common cause of illness in indigenous populations. Trans. R. Soc. Trop. Med. Hyg..

[29] Watt G., Strickman D. (1994). Life threatening scrub typhus in a traveler returning from Thailand. Clin. Infect. Dis..

[30] Silpapojakul K. (1997). Scrub typhus in the western Pacific region. Ann. Acad. Med. Singap..

[31] Corwin A., Sonderquist R., Suwanabun N., Sattabongkot J., Martin L., Kelly D., Beecham J. (1999). Scrub typhus and military operations in Indochina. Clin. Infect. Dis..

[32] Jiang J., Marienau K.J., May L.A., Beecham H.J., Wilkinson R., Ching W.M., Richards A.L. (2003). Laboratory diagnosis of two scrub typhus outbreaks at Camp Fuji, Japan in 2000 and 2001 by enzyme-linked immunosorbent assay, rapid flow assay, and Western blot assay using outer membrane 56-kD recombinant proteins. Am. J. Trop. Med. Hyg..

[33] Rodkvamtook W., Ruang-areerate T., Gaywee J., Richards A.L., Jeamwattanalert P., Bodhidatta D., Sangjun N., Prasartvit A., Jatisatienr A., Jatisatienr C. (2011). Isolation and characterization of *Orientia tsutsugamushi* from rodents captured following a scrub typhus outbreak at a military training base, Bothong District, Chonburi Province, central Thailand. Am. J. Trop. Med. Hyg..

[34] Sheehy T.W., Hazlett D., Turk R.E. (1973). Scrub typhus: A comparison of chloramphenicol and tetracycline in its treatment. Arch. Intern. Med..

[35] Koh G.C.K.W., Maude R.J., Paris D.H., Newton P.N., Blacksell S.D. (2010). Review: Diagnosis of scrub typhus. Am. J. Trop. Med. Hyg..

[36] Taylor A.J., Paris D.H., Newton P.N. (2015). A systematic review of mortality from untreated scrub typhus (*Orientia tsutsugamushi*). PLoS Negl. Trop. Dis..

[37] Philip C.B. (1948). Tsutsugamushi disease (scrub typhus) in World War II. J. Parasitol..

[38] Sayen J.J., Pond H.S., Forrester J.S., Wood F.C. (1946). Scrub typhus in Assam and Burma: A clinical study of 616 cases. Medicine.

[39] Smadel J.E., Woodward T.E., Ley H.L., Philip C.B. (1948). Chloromycetin in the treatment of scrub typhus. Science.

[40] Oaks S.C., Ridgway R.L., Shirai A., Twartz J.C. (1983). Scrub Typhus.

[41] Smadel J.E., Jackson E.B., Cruise A.B. (1949). Chloromycetin in experimental rickettsial infections. J. Immunol..

[42] Bailey C.A., Ley H.L. (1952). The treatment and prophylaxis of scrub typhus with antibiotics. Ann. N. Y. Acad. Sci..

[43] Smadel J.E., Ley H.L., Diercks F.H., Cameron J.A.P. (1952). Persistence of *Rickettsia tsutsugamushi* in tissues of patients recovered from scrub typhus. Am. J. Hyg..

[44] Berman S.J., Kundin W.D. (1973). Scrub typhus in South Vietnam: A study of 87 cases. Ann. Intern. Med..

[45] Brown G.W., Saunders J.P., Singh S., Huxsoll D.L., Shirai A. (1978). Single dose doxycycline therapy for scrub typhus. Trans. R. Soc. Trop. Med. Hyg..

[46] Olson J.G., Bourgeois A.L., Fang R.C.Y., Dennis D. (1981). Risk of relapse associated with doxycycline therapy for scrub typhus. Rickettsiae and Rickettsial Diseases.

[47] Watt G., Kantipong P., Jongsakul K., Watcharapichat P., Phulsuksombati D., Strickman D. (2000). Doxycycline and rifampicin for mild scrub-typhus infections in northern Thailand: A randomised trial. Lancet.

[48] Brown G.W., Shirai A., Jegathesan M., Burke D.S., Twartz J.C., Saunders J.P., Huxsoll D.L. (1984). Febrile illness in Malaysia—An analysis of 1629 hospitalized patients. Am. J. Trop. Med. Hyg..

[49] Alanis A.J. (2005). Resistance to antibiotics: Are we in the post-antibiotic era?. Arch. Med. Res..

[50] Kelly D.J., Richards A.L., Temenak J., Strickman D., Dasch G.A. (2002). The past and present threat of rickettsial diseases to military medicine and international public health. Clin. Infect. Dis..

[51] McClain J.B., Joshi B., Rice R. (1988). Chloramphenicol, gentamycin, and ciprofloxacin against murine scrub typhus. Antimicrob. Agents Chemother..

[52] Eaton M., Cohen M.T., Shlim D.R., Innis B. (1989). Ciprofloxacin treatment of typhus. JAMA.

[53] Raoult D. (2001). Antimicrobial activity against obligate intracellular bacteria. Trends Microbiol..

[54] Diene S.M., Abat C., Rolain J.-M., Raoult D. (2017). How artificial is the antibiotic resistance definition?. Lancet.

[55] Chosewood L.C., Wilson D.E. (2009). Section VIII-D: Rickettsial Agents. Biosafety in Microbiological and Biomedical Laboratories (BMBL).

[56] Barker L.F., Patt J.K., Hopps H.E. (1968). Titrations and neutralization of *Rickettsia tsutsugamushi* in tissue culture. J. Immunol..

[57] Wisseman C.L., Waddell A.D., Walsh W.T. (1974). In vitro studies of the action of antibodies on *Rickettsia prowazeki* by two basic methods of cell culture. J. Infect. Dis..

[58] Kelly D.J., Strickman D., Salata K., Hershey J. (1995). *Rickettsia tsutsugamushi* infection in cell culture: Antibiotic susceptibility determined by flow cytometry. Am. J. Trop. Med. Hyg..

[59] Raoult D., Drancourt M. (1991). Antimicrobial therapy of rickettsial diseases. Antimicrob. Agents Chemother..

[60] McOrist S. (2000). Obligate intracellular bacteria and antibiotic resistance. Trends Microbiol..

[61] Strickman D. (1996). Drug resistant scrub typhus. Proceedings of the 4th International Symposium on Public Health.

[62] Singh P. (2004). Scrub typhus, a case report: Military and regional significance. Med. J. Armed Forces India.

[63] Takahashi M., Murata M., Misumi H., Hori E., Kawamura A., Tanaka H. (1994). Failed vertical transmission of *Rickettsia tsutsugamushi* (Rickettsiales: Rickettsiaceae) acquired from rickettsemic mice by *Leptotrombidium pallidum* (Acari: Trombiculidae). J. Med. Entomol..

[64] Rosenberg R. (1997). Drug-resistant scrub typhus: Paradigm and paradox. Parasitol. Today.

[65] Traub R., Wisseman C.L., Jones M.R., O’Keefe J.J. (1975). The acquisition of *Rickettsia tsutsugamushi* by chiggers (trombiculid mites) during the feeding process. Ann. N. Y. Acad. Sci..

[66] D’Costa V.M., King C.E., Kalan L., Morar M., Sung W.W.L., Schwarz C., Froese D., Zazula G., Calmels F., Debruyne R. (2011). Antibiotic resistance is ancient. Nature.

[67] Olaitan A.O., Rolain J.M. (2016). Ancient resistome. Microbiol. Spectr..

[68] Perry J., Waglechner N., Wright G. (2016). The prehistory of antibiotic resistance. Cold Spring Harb. Perspect. Med..

[69] Sonthayanon P., Peacock S.J., Chierakul W., Wuthiekanun V., Blacksell S.D., Holden M.T.G., Bentley S.D., Feil E.J., Day N.P.J. (2010). High rates of homologous recombination in the mite endosymbiont and opportunistic human pathogen *Orientia tsutsugamushi*. PLoS Negl. Trop. Dis..

[70] Smadel J.E., Jackson E.B., Bennett B.L., Rights F.L. (1946). A toxic substance associated with the Gilliam strain of *R. orientalis*. Proc. Soc. Exp. Biol. Med..

[71] Oaks S.C., Ng F.K.P., Elwell M.R., Groves M.G., Lewis G.E. (1985). Pathology of toxic death in mice following intravenous injection of *Rickettsia tsutsugamushi* strain Gilliam: Examination by light and scanning electron microscopy. Jpn. J. Med. Sci. Biol..

[72] Liu Y.X., Feng D., Suo J.J., Xing Y.B., Liu G., Liu L.H., Xiao H.J., Jia N., Gao Y., Yang H. (2009). Clinical characteristics of the autumn-winter type scrub typhus cases in south of Shandong province, northern China. BMC Infect. Dis..

[73] Jeung Y.S., Kim C.M., Yun N.R., Kim S.W., Han M.A., Kim D.M. (2016). Effect of latitude and seasonal variation on scrub typhus, South Korea, 2001–2013. Am. J. Trop. Med. Hyg..

[74] Yoshikura H. (2017). Seasonality and geographical distribution of tsutsugamushi diseases in Japan: Analysis of the trends since 1955 till 2014. Jpn. J. Infect. Dis..

[75] Cho N.H., Kim H.R., Lee J.H., Kim S.Y., Kim J., Cha S., Kim S.Y., Darby A.C., Fuxelius H.H., Yin J. (2007). The *Orientia tsutsugamushi* genome reveals massive proliferation of conjugative type IV secretion system and host-cell interaction genes. Proc. Natl. Acad. Sci. USA.

[76] Nakayama K., Yamashita A., Kurokawa K., Morimoto T., Ogawa M., Fukuhara M., Urakami H., Ohnish M., Uchiyama I., Ogura Y. (2008). The whole-genome sequencing of the obligate intracellular bacterium *Orientia tsutsugamushi* revealed massive gene amplification during reductive genome evolution. DNA Res..

[77] Liao H.M., Chao C.C., Lei H., Li B., Tsai S., Hung G.C. (2016). Genomic sequencing of *Orientia tsutsugamushi* strain Karp, an assembly comparable to the genome size of the strain Ikeda. Genome Announc..

[78] Liao H.M., Chao C.C., Lei H.Y., Li B.J., Tsai S.E., Hung G.C., Ching W.M., Lo S.C. (2017). Intraspecies comparative genomics of three strains of *Orientia tsutsugamushi* with different antibiotic sensitivity. Genom. Data.

[79] Daugherty S.C., Su Q., Abolude K., Beier-Sexton M., Carlyon J.A., Carter R., Day N.P., Dumler S.J., Dyachenko V., Godinez A. Genome sequencing of Rickettsiales.

[80] Fleshman A.C., Mullins K.E., Sahl J.W., Hepp C.M., Nieto N.C., Wiggins K., Hornstra O’Neil H., Paris D., Dittrich S., Kelly D. (2017). Comparative pan-genomic analyses of *Orientia tsutsugamushi* demonstrates the unprecedented extent to which gene duplication and divergence drive genomic diversity. BMC Biol..

[81] Rolain J.M., Raoult D. (2005). Prediction of resistance to erythromycin in the genus *Rickettsia* by mutations in L22 ribosomal protein. J. Antimicrob. Chemother..

[82] Rolain J.M., Raoult D. (2005). Genome comparison analysis of molecular mechanisms of resistance to antibiotics in the *Rickettsia* genus. Ann. N. Y. Acad. Sci..

